# Fighting Cancer with Mathematics and Viruses

**DOI:** 10.3390/v9090239

**Published:** 2017-08-23

**Authors:** Daniel N. Santiago, Johannes P. W. Heidbuechel, Wendy M. Kandell, Rachel Walker, Julie Djeu, Christine E. Engeland, Daniel Abate-Daga, Heiko Enderling

**Affiliations:** 1 Department of Immunology, H. Lee Moffitt Cancer Center & Research Institute, Tampa, FL 33612, USA; Daniel.Santiago@moffitt.org (D.N.S.); Wendy.Kandell@moffitt.org (W.M.K.); Julie.Djeu@moffitt.org (J.D.); 2 Department of Integrated Mathematical Oncology, H. Lee Moffitt Cancer Center & Research Institute, Tampa, FL 33612, USA; Rachel.Walker@moffitt.org; 3 German Cancer Research Center, Heidelberg University, 69120 Heidelberg, Germany; j.heidbuechel@dkfz-heidelberg.de; 4 Cancer Biology PhD Program, University of South Florida, Tampa, FL 33612, USA; 5 National Center for Tumor Diseases Heidelberg, Department of Translational Oncology, Department of Medical Oncology, 69120 Heidelberg, Germany; 6 Department of Cutaneous Oncology, H. Lee Moffitt Cancer Center & Research Institute, Tampa, FL 33612, USA; 7 Department of Oncologic Sciences, Morsani College of Medicine, University of South Florida, Tampa, FL 33612, USA

**Keywords:** oncolytic virotherapy, combination therapy, mathematical model, immune system, cancer, immunotherapy

## Abstract

After decades of research, oncolytic virotherapy has recently advanced to clinical application, and currently a multitude of novel agents and combination treatments are being evaluated for cancer therapy. Oncolytic agents preferentially replicate in tumor cells, inducing tumor cell lysis and complex antitumor effects, such as innate and adaptive immune responses and the destruction of tumor vasculature. With the availability of different vector platforms and the potential of both genetic engineering and combination regimens to enhance particular aspects of safety and efficacy, the identification of optimal treatments for patient subpopulations or even individual patients becomes a top priority. Mathematical modeling can provide support in this arena by making use of experimental and clinical data to generate hypotheses about the mechanisms underlying complex biology and, ultimately, predict optimal treatment protocols. Increasingly complex models can be applied to account for therapeutically relevant parameters such as components of the immune system. In this review, we describe current developments in oncolytic virotherapy and mathematical modeling to discuss the benefit of integrating different modeling approaches into biological and clinical experimentation. Conclusively, we propose a mutual combination of these research fields to increase the value of the preclinical development and the therapeutic efficacy of the resulting treatments.

## 1. Introduction

In vitro and in vivo experiments are model systems of the real world, and given the understanding of the limitations of such approaches, study results are to be interpreted as simplifications that may not hold truth in the real world, which for cancer biology is ultimately the patient. Whilst there is no substitute for wet lab experiments, for many biological problems it is not only unfeasible but actually impossible to test all possible experimental conditions, population heterogeneities or drug combinations for an infinite amount of time. Mathematical models may synergize with experiments in helping analyze experimental data to iteratively inform subsequent experiments with the highest likelihood of advancing our knowledge [1]. Mathematics is a formal language that can be used to describe a complex, non-linear system to, amongst others, evaluate the mechanistic underpinnings of dynamic behaviors and predict long-term behavior in silico. If fitted to experimental data, mathematical models could identify appropriate parameters to estimate contributions of different mechanisms to simulated emergent dynamics. From that, sensitivity analysis may be deployed to investigate which mechanisms truly drive experimental outcome to identify promising treatment targets for example. For cancer biology, it may be cell migration rather than cell proliferation that drives an aggressive phenotype [2]. The construction of a mathematical model with given assumptions may reveal useful information such as unknown rates that may be found during a parameter search. For example, if the proliferation rate is known for a cancer cell line, the death rate may be approximated by a tumor growth model by modeling a net growth rate parameter. The fitting of experimental data to a math model may either confirm negligible impact or the necessity for a death/exhaustion rate to be part of the model. Once the fitting is done, a model may then help interpret observations. Here, conditions of the math model may help validate the model where confounding or confirming characteristics coupled with the experimental data help to refine or affirm the model, respectively. Finally, a mathematical model may predict outcomes, albeit a complicated task. At this stage, predicted outcomes might be rejected due to impossible or improbable biological conditions necessary for the outcomes (much like an imaginary solution is not considered for an algebraic equation when used to solve a word problem that necessitates a real solution). Elaborate discussions on parameter fitting and biological implications on their virotherapy models can be found elsewhere [3,4]. It is of importance to understand that a mathematical model, often informed by limited experimental data, can never prove a biological hypothesis to be right—but it may prove a hypothesis false or support its plausibility.

A myriad of work has been done on mathematical modeling in immunology and virology. Alan Perelson and colleagues published a series of articles on optimal strategies in immunology [5,6,7]. Such models helped identify the dynamics and time course of HIV infection [8,9,10] and response to treatment [11]. Mathematical principles of immunology and virology are summarized in an excellent textbook [12]. In this review, we aim to describe and discuss the contribution that integrated mathematical modeling could make to the advancement of oncolytic virotherapy (OVT)—with emphasis on the immunological aspects of OVT.

More than 100 years passed between the first reported oncolytic effect of a virus [13] and the approval of virotherapy for cancer, first in China (H101 by Shanghai Sunway Biotech, 2005) then later by the U.S. Food and Drug Administration and European Medicines Agency (T-VEC by Amgen, 2015 and 2016, respectively). The first reported cancer remission in the context of viral infection was described in 1904 for a woman with myelogenous leukemia after being infected with influenza [13] (almost three decades before influenza was found to be a viral infection). Organized efforts to unlock the potential anticancer effects of viruses have been under way since the 1940–1950s [14,15,16,17]. In a 1949 clinical trial of Hepatitis B virus applied to Hodgkin’s lymphoma, Hoster, Zanes, and Von Hamm noticed that 7 of 22 patients improved in the clinical aspect of the disease; a reduction of tumor volume was observed in 4 of 22 patients [14]. Therapeutic effects were found to rely on natural selective replication of viruses in tumor cells. Moreover, mechanisms of action are being investigated to further develop OVT. Modes of effective OVT may include any individual or combination of cellular lysis, apoptosis, and innate or adaptive immune responses [18]. Various genetically engineered oncolytic viruses (OVs) are available, and the diversity of OVT combination regimens has grown rapidly in recent years, especially with emphasis on personalized cancer therapy. In the hope of guiding and optimizing these more complex therapies, mathematical models are deployed to understand the key mechanisms underlying the complex biological interactions. Having quantitative models in place that can simulate effective OVT could greatly reduce time and effort in the search for optimal OVT for subpopulations of cancer patients.

## 2. Mathematical Modeling of Tumor Growth

Oncolytic efficacy depends on tumor growth dynamics, of which mathematical modeling has a long history [19,20]. Differential equations are commonly used to describe the mechanisms that govern change in tumor cell number, or tumor volume:

(1) $$\frac{dc}{dt}=r\times c$$

where the variable *c* represents the number of cells, *dc* denotes the change (difference, *d*) in cell number during a specific time interval, *dt*. The rate of change *r* describes the net change of the cell population. In the simplest case, r could be a constant number (such as *r* = 0.1), which describes the rate at which the number of existing cells increases. This will lead to exponential growth as in each time interval *dt* the number of cells increases by *r* × *c*. Intuitively, if *r* is a negative number, the population will decay. Experimental measurements such as cell numbers in vitro or volumes in vivo may help identify the value of such parameter *r*. Of note, *r* itself could be a function that describes in more detail how the number of cells could change, for example *r* = *f* − *g*, where *f* could describe the rate at which cells divide per unit time, and *g* the corresponding rate of cell death. The results of net growth is identical to only using the population net growth rate *r*, but the separation of cell division and cell death events may become very important in virology where viral dynamics are strongly influenced by those processes. Different growth laws have been developed and successfully fit to experimental and clinical tumor volume data. These growth laws can be as simple as above-discussed exponential growth. More complicated models may include increasingly complex biology. Initial exponential growth at low densities, when most cells have access to ample resources, decelerates when cells at the core of the tumor become growth-arrested. This is largely due to limited space and exhausted intratumoral nutrient supply as resources are consumed by cells closer to the tumor surface [21,22,23]. This established the notion of a tumor carrying capacity (*K*) as the maximum cell number that can be supported by a given environment [24]. The rate at which tumor growth saturates as the cell number approaches its carrying capacity can be shaped differently including linear (logistic growth; *f* = *a* × (1 − *c*/*K*)) and logarithmical (Gompertzian; *f* = *a* × ln(*K*/*c*)) functions, etc. A tumor carrying capacity may evolve with changing oxygen and nutrient supply through tissue vascularization, removal of metabolic waste products, and evasion of immune surveillance [25,26]. Then, carrying capacity itself will become a variable, i.e., *dK*/*dt*, whose rate of change can be described with a differential equation [25].

The most likely growth law for a specific tumor can be obtained by fitting the different models to experimental/clinical data and comparing the regression results [27]. The carrying capacity of the tumor may be more important as cancer cells multiply and the tumor grows; consequently, time constraints of the experiment or mathematical modeling may influence the decision to incorporate a tumor carrying capacity. Ordinary differential equation (ODE) systems are typically used for relatively simple models of growth dynamics. In a recent study, Murphy and colleagues showed that different tumor growth models fit retrospective experimental and clinical data equally well, but forward predictions in time may significantly vary [28]. There is a need to identify the most applicable growth dynamics [29], with explicit consideration of available data and the number of undetermined mathematical model parameters and their identifiability [30,31,32]. The Akaike information criterion (AIC) is often utilized to correlate model complexity with fit to data. This penalizes models with too many degrees of freedom and only marginal improvements in data fit [33].

While most examples in this review will be ODE systems, mathematical modeling of tumor growth and oncolytic virotherapy may also be described by partial differential equations (PDE) or agent-based model (ABM) systems. While ODE systems describe quantities over time, PDE systems describe quantities over time and relative dimension in space. A spatially explicit PDE system would become necessary when the biological effects are predominantly space-dependent (such as diffusion of cancer drugs or viral penetration into a tumor) and cannot be averaged in an ODE model (see de Pillis et al. [34]). Agent-based models (or single cell models; each cell is an individual “agent”) may be used to simulate individual cell behaviors and cell–cell or cell–environment interactions that propagate to produce emerging population-level dynamics. From such an approach, one can exclude or identify likely cellular mechanisms that underlie observed complex system-level behavior. See Wang et al. [35] for a recent review on ABM models of tumor cells and Wodarz [36] for a review on overall approaches to OV dynamics.

## 3. Viral Life Cycle

Viral entry into a host cell, replication within the cell and finally release of progeny particles is often referred to as the “life cycle” of a virus. Cellular requirements for the completion of the viral life cycle compare to the hallmarks of cancer [37]. Both processes benefit from pro-mitogenic, antiapoptotic and metabolic alterations promoting cell survival, proliferation and protein biosynthesis. Inflammation provides further stimuli. Induction of angiogenesis improves supply of nutrients and oxygen and furthermore allows spread of both viruses and tumor cells. The disruption of innate antiviral pathways, namely of the interferon (IFN) response, by mutations or virulence factors, is another important common mechanism of both viral infection and tumorigenesis. Viral infection and malignant transformation therefore share important signaling pathways and core elements required for successful progression (reviewed in detail in [38]).

Consequently, the first known oncogenes, amongst them the protein kinase-encoding genes in Rous sarcoma virus (*v-Src* [39,40]) and Abelson murine leukemia virus (*v-Abl* [41]), were found to be acquired from viruses. There are several ways in which viral infection can support malignant transformation. Both expression of oncogenic viral proteins, e.g., E6 and E7 in human papillomavirus (HPV)-associated cancers [42,43], and insertional mutagenesis—as observed in a gene therapy trial with retroviral vectors [44]—can provide a survival advantage to infected cells by promoting genetic instability, activating mitogenic signaling pathways and inhibiting apoptosis. Persistent inflammation as a result of viral infection also contributes to tumorigenesis in several cancer types such as hepatocellular carcinoma induced by chronic infection with hepatitis B or C viruses (reviewed in [45]).

On the other hand, anecdotic observations of cancer remissions associated with viral infections have been reported historically [13,46], indicating a potential for using these “culprits” as a strategy to cure cancer. This approach is also linked to the close resemblance of mechanisms required for viral replication and malignant transformation. Facilitation of productive viral infection by changes in cellular signaling, metabolism and innate immune responses in the course of malignant transformation has been referred to as “phenotypic complementation” [38]. Providing cellular requirements for productive infection can thereby enable viruses that are not able to replicate within healthy tissue to selectively infect and kill tumor cells.

Such viruses, which can either have a natural tropism towards cancer cells or be genetically engineered to enhance tumor-specific replication, are termed OVs and can be found throughout different virus classes [47]. In contrast to oncogenic viruses that cause latent infections allowing host cells to survive and accumulate mutations, OVs usually have a lytic replication cycle leading to the death of infected cells. Oncolytic virotherapy is a promising approach to treat cancer by making use of these agents, relying on a variety of mechanisms of action differing from those of conventional treatment options such as surgery, chemotherapy and radiotherapy.

A unique feature of OVT is the amplification of the agent within the tumor, increasing the therapeutic potential of the initially applied dose [38]. Killing of infected cells typically occurs by either extensive budding of viral progeny or expression of viral proteins on the cell surface and subsequent fusion with neighboring cells, both finally resulting in bursting of the host cell, i.e., oncolysis [48]. The unique mechanisms of OV cytotoxicity generally do not completely rely on intrinsic cell death programs, thus providing potential to overcome typical forms of treatment resistance observed in chemotherapy and radiotherapy [38]. Both viral amplification and the individual modes of oncolysis are important factors that require consideration in the mathematical modeling of OV infection.

**Figure 1:**
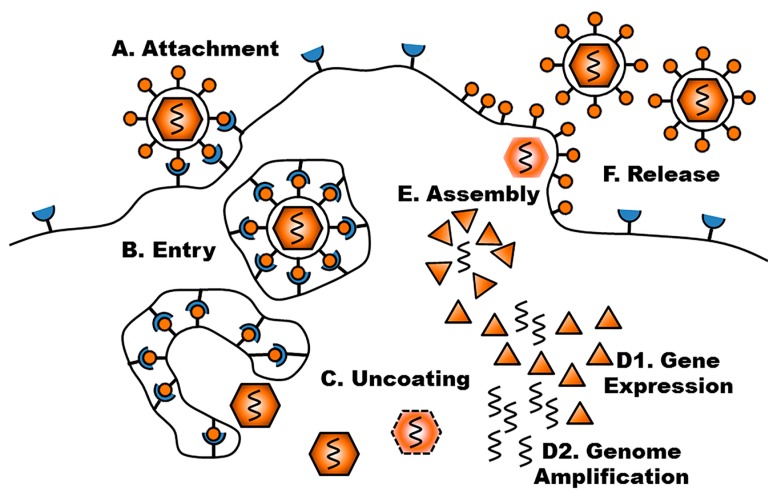
Schematic representation of the reproductive cycle of viruses. After successful attachment to a cell entry receptor (**A**), entry via membrane fusion or endocytosis (**B**), and uncoating of nucleic acids (**C**), viral gene expression (**D1**) and genome amplification (**D2**) are initiated. These processes can be complex and may include reverse transcription of the genome, shuttling to the nucleus, and further processing and modification of generated nucleic acids and proteins. Assembly (**E**) and subsequent release (**F**) of viral progeny complete the viral “life cycle”. Each of these steps contributes to (tumor) cell specificity of a particular virus as well as its replicative and cytolytic potential and, depending on the scientific question, may need to be considered in mathematical modeling.

The sequential steps in the reproductive cycle of a virus (Figure 1) can differ substantially between individual viruses and influence their rate of cell killing and spread, accordingly affecting their oncolytic potential. For a productive infection, one or more virus particles must enter the host cell. This process differs between classes of viruses, especially regarding the presence or absence of a viral membrane envelope. Attachment to cells is mediated via viral surface proteins targeting molecules accessible on cell membranes. Adaptation to cell entry receptors contributes to viral tropism. The host cell range can be broad, as in the case of vesicular stomatitis virus (VSV), which binds to low-density lipoprotein receptor (LDL-R) via the vesicular stomatitis virus G glycoprotein (VSV-G) [49], or more limited: wild type measles virus requires interaction of the hemagglutinin (H) protein with signaling lymphocyte activation molecule (signaling lymphocytic activation molecule (SLAM), cluster of differentiation (CD) 150) [50] or the adherens junction protein Nectin-4 [51] for entry and is restricted to immune and epithelial cells, which express these molecules, respectively. For some viruses, the host cell specificity can be modified by genetic engineering. Serial passaging of wild type measles virus has resulted in the generation of live attenuated vaccine strains [52]. These have adapted to usage of the complement-regulatory protein CD46 [53,54,55], which is frequently overexpressed on tumors, in addition to its natural tropism. Point mutations in the *H* gene can abrogate binding to these receptors, and introduction of transgenes encoding single chain antibodies or receptor ligands can be applied to retarget measles virus [56,57,58]. Pseudotyping with attachment proteins of different virus families is another tool for retargeting of viruses which can be applied e.g., to broaden the host cell range of lentiviral vectors via VSV-G (reviewed in [59]) or measles virus glycoproteins [60].

Upon attachment, the viral particle passes the host cell membrane in a process termed penetration, involving different mechanisms depending on the virus type. Measles virus and most other members of the family *Paramyxoviridae* require interaction of two distinct surface proteins for entry. Binding of the H protein to a cell entry receptor induces a change in the structural conformation of the F (fusion) protein, leading to an approximation of viral particle and host cell. This finally results in membrane fusion, allowing entry of the viral particle into the cell (Plattet et al. [61], and Lamb and Parks [62]). Endocytosis provides an alternative route of cell entry. Acidification of maturing endosomes can induce conformation changes in viral attachment proteins to promote membrane approximation and fusion [63]. An example for this mechanism is the H protein of influenza viruses, which are also studied as potential oncolytic vectors [64,65,66]. Non-enveloped viruses omit the need for membrane fusion and rely on endocytic pathways for entry (reviewed in [67]).

In line with the heterogeneity of genome sizes among OV, ranging from approximately 5 kb of oncolytic parvovirus [68] to 300 kb of oncolytic vaccinia viruses [69], the number of genes and complexity of their regulation also differs greatly. For more detailed information on genome organization and replication of particular viruses, please refer to comprehensive reviews, e.g., for herpes simplex virus [70], poxvirus [71] and adenovirus [72]. Before amplification and expression of the viral genome can take place, it must be made accessible by uncoating. For many viruses, transfer of viral nucleic acid into the nucleus is furthermore required to make use of the genome amplification and transcription machinery of the host cell. Some viruses, including paramyxoviruses, which have their negative strand RNA genome packaged into so-called ribonucleoprotein complexes, harbor the enzymatic machinery necessary for transcription into mRNA [73].

Viral gene expression and genome amplification can be tightly regulated, e.g., by promoter elements and pre-messenger RNA (mRNA) processing. This enables restriction to certain cell types and adaption to changes in the phenotype of the host cell, such as the differentiation of HPV-infected epithelia that is necessary for the virus to complete its life cycle (see reviews [74,75,76]). The presence of immediate early, early and late genes in herpes viruses [77] relates to different phases of infection and ensures efficient replication before cell lysis. The time lapse between viral entry and progeny release, called “eclipse phase”, plays an important role in viral replication kinetics [78].

With regards to the safety aspects of oncolytic virotherapy, such post-entry mechanisms can be exploited to enhance tumor specificity or interfere with viral replication. Introduction of target sites for microRNAs (miRNAs) downregulated explicitly in malignant cells can prevent viral replication in normal tissue [79,80,81]. Exchanging furin cleavage sites necessary for cleavage and activation of viral proteins with target sequences for tumor-specific matrix metalloproteinases represents another strategy to prevent off-tumor toxicity [80,82]. Riboswitches, which undergo self-cleavage upon ligand administration, can be applied as off-switches for viral replication [83]. Tumor specificity can also be conferred at the level of protein translation, by inclusion of polyadenylation signals in the mRNA of key viral proteins [84,85]. Depending on the virus, DNA of viral origin might be integrated into the host cell genome, potentially inducing (epi-)genetic deregulation [86] and mutagenesis (reviewed in [87]). In contrast, RNA viruses are obligatory cytoplasmic and cannot integrate into the host cell genome, contributing to a favorable safety profile regarding their use as oncolytic agents.

Once the viral genome is replicated and structural proteins are expressed, assembly of viral progeny is initiated and finally mature particles leave the host cell via budding, pore formation and/or bursting of the cell. Some viruses, including measles virus, can infect neighboring cells without the necessity of forming extracellular particles by exploiting cell–cell contacts [88] and by display of viral attachment and fusion proteins on the host cell surface [89]. 

Oncolytic virus replication might be regarded in a very basic sense as releasing infectious particles via killing of infected cells, which makes it an appealing target for mathematical modeling. However, as viral infection is a highly complex, virus-specific, multistep process, an understanding of the underlying biology of virus–host interactions is required for careful consideration of appropriate modeling approaches. As a basis for a mathematical model of oncolytic virotherapy, a model of viral infection, uninfected cells becoming infected cells, is first considered.

## 4. Mathematical Modeling of Infection: Susceptible and Infected Model

Viruses are infectious agents that rely on a living host cell to replicate. Infectious disease modeling has an extensive history in mathematics to simulate viral spread and cytotoxic effects [90]. Host cells are divided into susceptible (uninfected, S) and infected (I) cells, where C (number of total tumor cells) = S + I. (Note that “C = S + I” does not include resistant cells without receptors for viral entry or stromal cells not targeted by OV.) Such SI models evolved from ecological population dynamics, e.g., food chain [91] (predator, prey, and top-predator) first used for infectious diseases (reviewed in [92]) then eventually for viruses as early as 1996 by Nowak and Bangham [93]. In 1995, Gatenby modeled cancer as a population competing with normal cells [94]. These early models have contributed to the evolution of the mathematical modeling of oncolytic virus therapy [36,95]. Nowak, Perelson, and others [12,93,96,97,98] used models that assume constant target cell production. Wodarz first described OV dynamics by adapting basic virus dynamics models, assuming a density-dependent proliferation of target (tumor) cells [99], as well as tumor-specific immune responses (virus-specific cytotoxic T lymphocytes) [99,100]. Others have expanded on this basis such as Dingli [101] and Biesecker [102].

**Figure 2:**
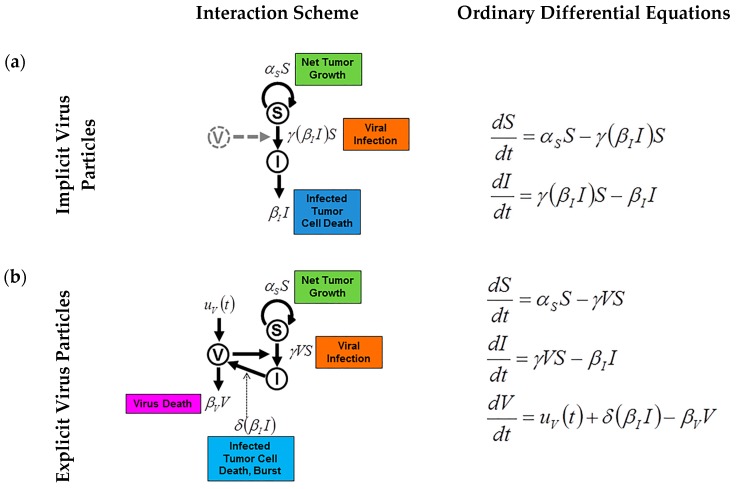
Simple susceptible (S) and infected (I) models with implicit (**a**) and explicit (**b**) model representations of a virus population. The *β_I_I* term (**a**) represents the population of dying infected cells. These infected cells release virus particles—not represented—upon death (also called burst [103]), hence, directly affecting the number of susceptible cells, which may become infected, *γ*(*β_I_I*)*S*. In explicit virus systems (**b**), the *β_I_I* term is commonly described as explicitly affecting the number of virus particles (*δ*) as opposed to the virus being modeled implicitly from its effects on the *S* and *I* populations. Consequently, the viral infection term is *γVS*. The term *U_V_*(*t*) simulates the effect of oncolytic virotherapy, a number of virus particles administered at time *t*. Novozhilov et al. warns that mass-action, the assumption that the rate is proportional to the product of the variables, may only be acceptable when the *S* and *I* populations are close in value, *S* ≈ *I* [95].

Experimental and clinical observations often present data for the tumor volume, which can be converted into a total number of tumor cells, but typically are not of sufficient resolution to classify subpopulations of infected and non-infected tumor cells. It may be possible to capture viral infection dynamics with an implicit representation of virus particles [104] (Figure 2a), though more complicated models may require an explicit population of virus to be modeled [105] (Figure 2b). It should be noted that a model with an implicit virus representation may introduce an OV treatment by changing a quantity or proportion of uninfected cells to infected cells at the time of treatment (see *f*_0_ in supplemental information of [104]). Using an implicit representation limits the number of variables (and parameters) to the model system. However, this formulation is only applicable if the turnover rate of free virus particles is much faster compared to infected cells. Alternatively, the free virus population must be modeled explicitly as a time-dependent variable, which requires the inclusion of more parameters, some of which may be challenging to identify.

A basic model for simulating viral infection has been previously discussed as a system of ODE [104]. In this simple SI model with implicit virus particles, the uninfected, susceptible tumor cells (variable *S*) may grow with rate α_S_ (exponential tumor growth). With viral infection rate *γ*, uninfected cells become infected (variable *I*), and infected cells die with a fixed rate, *β_i_*. A schematic of this simple model and the corresponding equations are shown in Figure 2a. Modeling the actual virus population introduces a new variable, *V*, with an arrival rate (first exposure, therapy, etc.) and intrinsic death. Infection is then explicitly modeled by the interaction of susceptible cells with virus particles, *S* × *V*, which occurs with rate *γ*.

## 5. Modes of Action in Oncolytic Virotherapy

**Figure 3:**
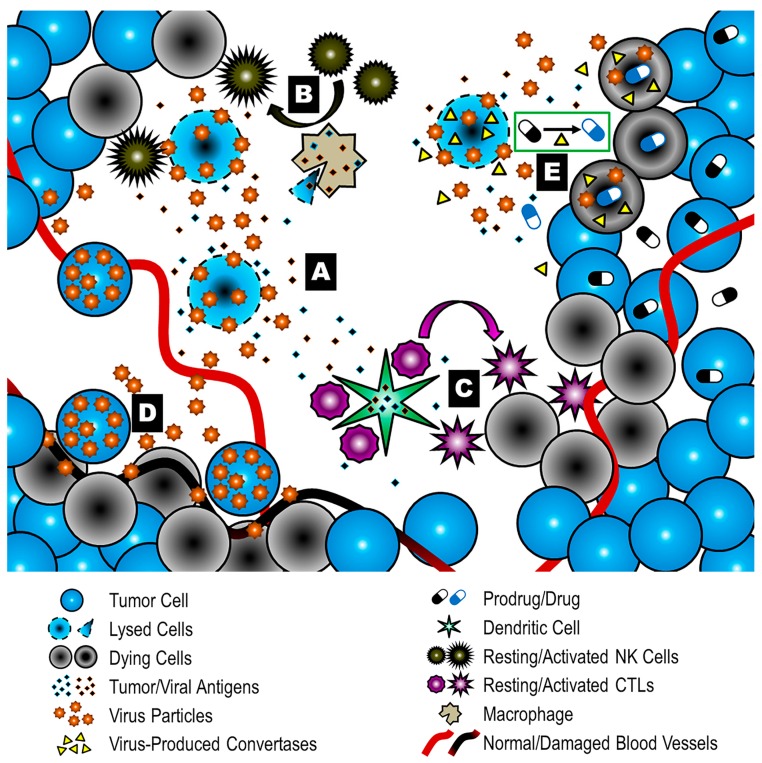
Multi-mechanistic modes of action of oncolytic virotherapy. Direct lytic replication (**A**) can be supported by additional antitumor processes in the context of oncolysis, e.g., immunogenic cell death and subsequent innate (**B**) and adaptive (**C**) antitumor immune responses, and endothelial targeting (**D**). This complexity can be exploited and expanded by genetic modifications and combination therapies. One approach is encoding prodrug convertases in the viral genome, which mediate local activation of systemically applied prodrugs (**E**). CTLs: Cytotoxic T lymphocytes; NK: Natural killer.

In recent years, it has become apparent that direct tumor cell destruction via lytic replication is not the only mode of action contributing to the efficacy of oncolytic treatment (Figure 3), and might even be only a minor determinant of treatment success [106]. More importantly, oncolysis represents a form of immunogenic cell death, resulting in the release of potent immune-stimulatory molecules, including cytokines, pathogen-associated and damage-associated molecular patterns (PAMPs and DAMPs, respectively) and tumor-associated antigens (reviewed by Workenhe et al. [107]). This can induce recruitment of cells of both the innate and the adaptive immune system, potentially leading to systemic antitumor immunity. Tumor debulking by lytic replication can be seen as the initial step to reversing immune evasion mechanisms in the tumor microenvironment and evoking tumor-targeting processes [106]. Modes of action of tumor cell killing during the immune response have been summarized by Cassady and colleagues as cytokine-induced apoptosis, cytotoxicity of innate immune cells, and antigen-specific tumor cell lysis by T cells [18].

Viral infection is detected intracellularly via pattern recognition receptors (PRRs) such as retinoic acid-inducible gene 1 (RIG-1), which induces expression and secretion of pro-inflammatory cytokines and type I IFNs upon binding of viral RNA (reviewed by Barik et al. [108]). This typically leads to an antiviral state in surrounding cells, but can also result in killing of uninfected tumor cells by cytokines such as tumor necrosis factor α (TNFα) [109]. Innate immune effectors encompass natural killer (NK) cells, which are able to induce lysis of tumor cells or virally infected cells (reviewed in [110]), and phagocytic cells such as neutrophils and macrophages.

In the course of phagocytosis or tumor cell lysis, uptake of tumor-associated antigens (TAAs) by antigen-presenting cells (APCs) such as dendritic cells (DCs) is essential for the induction of an adaptive immune response [111,112]. Cross-presentation of such antigens on major histocompatibility complex I (MHC-I) molecules to CD8^+^ T cells in lymph nodes, followed by cytokine-mediated stimulation, is necessary for activation and expansion of tumor-specific cytotoxic T lymphocytes (CTLs). This is supported by differentiation of CD4^+^ T cells towards a type 1 T helper (Th1) phenotype, indicated by release of interleukin (IL)-2 and IFN-γ [113] (reviewed by Farrar et al. [114]). Another crucial aspect in terms of timing, efficacy and durability of antitumor immune responses is the development of memory and effector cell subsets [115,116].

Polarization of macrophages in the tumor microenvironment towards a pro-inflammatory (M1) rather than immunosuppressive phenotype (M2) further contributes to tumor destruction [117]. Inflammatory and other adjuvant stimuli provided by viral infection play a major role in promoting such antitumor effector functions. In their absence, innate sensing of tumor cells and TAA presentation will instead induce tolerance mechanisms, e.g., by regulatory T cells (Tregs) [118]. It has been shown that established TAA-tolerance of the immune system can be overcome by an intratumoral injection of a non-replicating, immunogenic adenovirus [119]. An OV-based combination regimen aiming at enhancing immunogenic cell death was also effective in breaking immune tolerance [120].

Based on this understanding, current development of novel oncolytic vectors focuses not only on enhancing lytic replication, but also on harnessing the immune response, for example by the introduction of transgenes encoding cytokines [121,122], checkpoint inhibitors [123], ligands of T cell co-stimulatory receptors [124], bispecific T cell engagers [125,126,127] or tumor antigens [128,129,130], respectively, into the viral backbone. Combination therapies represent another approach to support immune responses to OV treatment, including additional application of cytokines [131], immune checkpoint inhibitors [132,133] or chemotherapeutics [134].

Immune responses to OV treatment are highly complex and can also prevent successful therapy by limiting viral infection, depending on context and timing. For this reason, even immunosuppression might be beneficial prior to OV treatment to enhance viral replication and spread [135,136].

In addition to making use of the immune system, bystander killing of non-infected tumor cells can also be increased by using virus-encoded prodrug convertases [137]. Upon infection of tumor cells, the transgene is expressed at the tumor site. Non-toxic prodrugs are applied systematically and converted locally into a chemotherapeutically active compound, thereby minimizing toxicity to healthy tissue [138].

Targeting of tumor vasculature by oncolytic viruses has been observed [139,140] and was recently explained by suppression of cell-intrinsic antiviral mechanisms via vascular endothelial growth factor (VEGF) in the tumor microenvironment [141]. This could add to the antitumor potency of oncolytic virotherapy by inducing subsequent nutrient deprivation and hypoxia, but also impair viral delivery to the tumor site, warranting careful assessment prior to manipulating the antiendothelial potential of an oncolytic vector to enhance clinical benefit. Sunitinib-mediated inhibition of the VEGF receptor represents a potential approach for combination therapy [136,142]. 

There is emerging evidence for modes of action of OVT that go beyond the killing of infected cells by lytic replication. Models of viral spread and treatment outcome should appropriately account for these modes to enable accurate predictions. In a mathematical model, these modes first translate into the speed of growth functions, delays due to the viral life cycle, and mathematical descriptions of physical viral tropisms (manner of infection) as discussed in Section 6.

## 6. Modeling Specific Mechanisms of Action

Wodarz and Komarova have examined fast and slow classes of viral growth with biological interpretations of non-solid (“liquid”) tumors and solid tumors, respectively [104]. Fast viral growth is indicative of a well-mixed system, where there is little to no restriction on the viral infection rate. This represents some in vitro experiments as well as non-solid tumors. The slow class viral growth is necessary when modeling solid tumors due to spatial penetration dynamics. These fast and slow infection terms may be formulated in a variety of ways, which are discussed thoroughly in [104]. Examples of such viral spread terms include $\frac{\left(\varepsilon +1\right)S}{\left(S+I+\varepsilon \right)}$ (fast) and $\frac{\left(\varepsilon_{1}+1\right)\left(\varepsilon_{2}+1\right)S}{\left(S+\varepsilon_{1}\right)\left(I+\varepsilon_{2}\right)}$ (slow), where *S* and *I* are the number of susceptible uninfected and infected cells, and *ε*, *ε*_1_ and *ε*_2_ are empirical coefficients to describe saturation kinetics [143,144]. Wodarz and Komarova have stated that it can be difficult [145] to fit these models to experimental data [3,104,146], such that arbitrary viral expression terms may have no basis of any biological mechanism. That is, when fitting models to experimental data, in vitro experiments of a few days or in vivo experiments of about 30 days, these functions may all fit similarly well, and it is not until well after the time period of experimental data will models differ due to the different infection terms. An intratumoral injection of viral therapy into a solid tumor, for example, places a high concentration of virus particles in one specific location. Infection rate would be highest at the interface of this high virus concentration and the adjacent tumor cells, while almost no infection would take place in the tumor periphery. A spatially explicit partial differential equation (PDE) system may be employed for a more realistic depiction of spatial propagation of the virus after initial infection. An example of such a system is discussed in detail by Jacobsen et al. [147].

For modeling purposes, the time period from viral entry into tumor cell to tumor cell burst (“eclipse phase”) may be important [148]. In cell adhesion assays, for example, time periods that coincide with the viral replication cycle elapse between the time of oncolytic virus treatment of a cell line and observable effect [149]. Such delayed responses can be simulated mathematically by a delay differential equation (DDE) [150]. Further complexities may be added to a mathematical model to explicitly account for the interplay between multiplicities of viral infection and the antiviral states mediated by interferon [148,151,152] as a cellular response to viral infection. Of course, the model would require additional distinctions of antiviral and non-antiviral states [151] for uninfected cells (for example, see [90]).

The potential immune response to an oncolytic virus adds further complexity. In 2011, Eftimie et al. [4] reported a model based on an oncolytic immunotherapy study presented by Bridle et al. [129]. In this study, an adenovirus (Ad) vaccine was first used for immunization [153,154] before a VSV was used for oncolytic treatment against intracranial and systemic tumors (B16-F10 and CT26 cells, established in C57BL/6 and BALB/c mice, respectively). The Ad and VSV were designed to express the same TAA as the tumor cells. Bridle et al. [129] demonstrated that VSV increased a pre-existing antitumor immune response by shifting the immune response from viral antigens to tumor antigens. Further, a distinction between two compartments, lymphatic and peripheral (tumor) tissues, enabled simulation of a systemic effect of the OV on the immune system. Specifically, the recruitment and infiltration of effectors to the peripheral tissue was modeled due to an antiviral response in the lymphatic tissue. Mathematical analysis of model dynamics can identify a possible “tumor only” state (tumor without virus) when: (1) the inactivation rate of peripheral effectors is high; or (2) when the tumor is aggressive (high net tumor growth rate). While these observations may be biologically obvious, such analysis helps validate mathematical descriptions of the complex system as such conclusions are not built in a priori. More complex analyses help identify conditions under which the three equilibrium states of tumor-free, tumor only, and coexistence of tumor with virus are obtained. In this particular example, VSV must persist for a long time to achieve a tumor-free state. The model suggests, again quite intuitively and thus confirming model applicability, that oncolytic viruses with a higher half-life or better replication rate yield increased efficacy.

**Figure 4:**
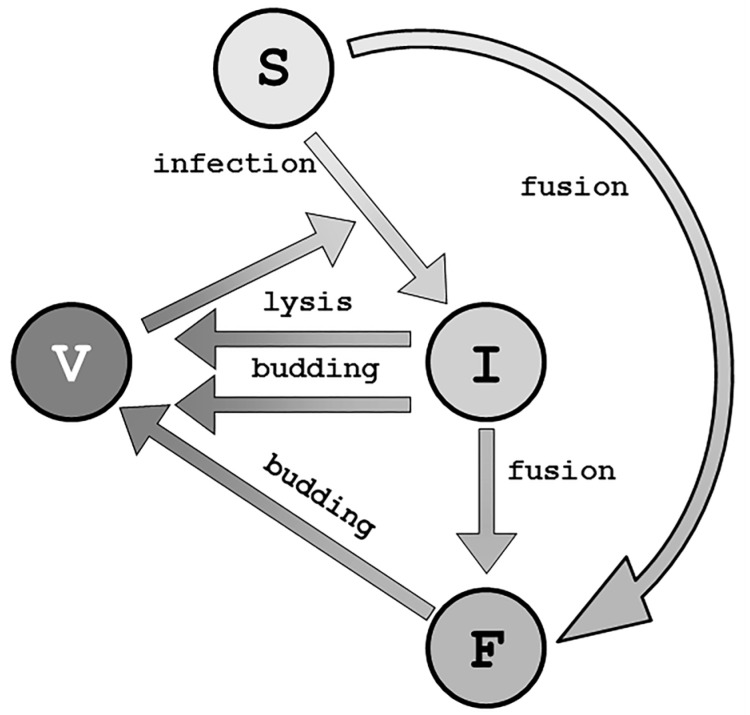
Schematic of general virus–host interactions. S: Susceptible cell; I: Infected cell; F: Fused cells; V: Virus. Each arrow can be described mathematically with a rate constant or more complex functions to describe different mechanisms of action (adapted from [147]).

Bajzer and Dingli as well as Jacobsen and Pilyugin added a syncytia-forming fusion and budding mechanisms to lysis in a PDE model (Figure 4) that may be tailored to a particular viral mode of action [3,101,102,155]. Jacobsen and Pilyugin built upon previous PDE systems from Wu and Friedman et al. [156,157]. These models only allowed budding as a mechanism for viral particle production from syncytia, assuming that no apoptosis occurs from fused cells [158]. Recent reports suggest that this may not be the case [159,160,161,162,163,164,165], depending on the virus; adjustments during model development and fitting can resolve such issues. In their model, Jacobsen and Pilyugin found that an increase in burst size would allow for tumor control. However, different fusion (formation of syncytia) rates, $\bar{\rho }$, predict the outcomes of an inert virus $\left(0.1<\bar{\rho }<0.5\right)$ or control of tumor growth $\left(\bar{\rho }<0.1,\bar{\rho }>0.5\right)$. The authors hypothesized that at a low $\bar{\rho }$ yields a governing lysis rate leading to control of the tumor, and at high $\bar{\rho }$, syncytia reportedly leads to fusion of all tumor cells and an exponential decay of tumor cells. Fusion rates between these two categories kill neighboring tumor cells but render remaining virus particles ineffective.

## 7. Current Developments in Oncolytic Virotherapy

A total of 81 clinical trials for oncolytic viruses were listed on ClinicalTrials.gov [166] compared to only six studies 10 years ago, demonstrating the rapid development of the field. Compared to the first approaches to using viruses for cancer treatment, in some cases by application of infectious body fluids, research has made huge progress due to a deeper understanding of underlying virological processes and the possibility of genetic engineering (reviewed in [167]).

The most prominent example of the clinical translation of oncolytic viruses is talimogene laherparepvec (T-VEC, trade name Imlygic^TM^), a genetically modified herpes simplex virus type I. In addition to gene knockouts for enhanced tumor specificity and immunogenicity [168], T-VEC encodes the cytokine granulocyte-macrophage colony-stimulating factor (GM-CSF) for increased infiltration and activation of myeloid cells to support antitumor immunity [169]. As the first—and so far only—oncolytic therapeutic, T-VEC has been granted market approval in the United States in 2015, and shortly afterwards also in Europe, for the treatment of advanced melanoma. Investigations of its use in other malignancies and in combination therapies, especially with immune checkpoint inhibitors, are underway (trial examples for breast cancer: NCT02658812; pancreatic cancer: NCT03086642; combination with radiation: NCT02453191; combination with Nivolumab and Pembrolizumab, respectively: NCT02978625 and NCT02965716). The phase III study that led to approval resulted in a significantly enhanced durable response rate and a higher median overall survival in the patient arm treated with T-VEC compared to patients receiving GM-CSF [170]. Importantly, intratumoral injections of T-VEC have led to remissions of uninjected, distant lesions, indicative of the induction of systemic antitumor immune responses. CD8^+^ CTLs were shown to play a major role in mediating these effects. However, the exact mechanism of action of T-VEC has not yet been fully elucidated [171].

Another prominent example of OV is oncolytic vaccinia virus JX-594 (pexastimogene devacirepvec, Pexa-Vec), which has advanced to a phase III trial for the treatment of hepatocellular carcinoma (NCT02562755). JX-594 is thymidine kinase-deficient, increasing tumor cell specificity, and, like T-VEC, encodes GM-CSF for enhanced antitumor immune activation. In addition, a transgene encoding β-galactosidase allows for analysis of viral replication [172,173,174]. A phase II study in hepatocellular carcinoma showed an acceptable safety profile and dose-dependent survival benefit after intratumoral injection of JX-594 [175]. Viral replication and transgene expression were verified by measuring genome concentrations in blood and by detecting GM-CSF and antibodies against β-galactosidase, respectively [175]. Tumor responses were observed in both injected and uninjected lesions [175].

MV-NIS, an oncolytic measles virus derived from a live attenuated vaccine strain encoding human thyroidal sodium iodine symporter (NIS), entered clinical trials for application in several malignancies including multiple myeloma [176] and ovarian cancer [177]. The NIS transgene allows for imaging of infected cells via intracellular accumulation of ^123^I and can also be used for enhanced tumor cell killing by radioactive ^131^I [178,179]. An important aspect of oncolytic measles virotherapy remains the prevalence of neutralizing antibodies against measles virus surface glycoproteins, which cannot be sufficiently addressed by retargeting of the *H* gene [180]. Although a much-noticed case of durable complete remission of disseminated multiple myeloma has been observed upon systemic treatment with MV-NIS in a phase I study [181], this patient had unique characteristics favoring therapeutic success: a specific gene signature indicating sensitivity to OVT was detected by sequencing [182] while anti-measles virus antibody titers were absent in blood serum. However, there are means to overcome antibody neutralization of oncolytic agents: polymer coating [183], pseudotyping [184,185], complement inhibition [186] and usage of infected cells as virus carriers [187,188,189,190].

In addition, viruses that are non-pathogenic for humans may be used to reduce the prevalence of pre-existing immunity within the population. A genetically attenuated strain of the Maraba rhabdovirus [191], which had originally been isolated from Brazilian sand flies [192], is currently being tested in a prime-boost setting after immunization with an adenovirus (NCT02285816). Both viruses were genetically modified to encode the TAA MAGE A3 in order to evoke a strong tumor-specific immune response rather than boosting anti-viral responses [130,193]. Rodent parvovirus H-1 (H-1PV/ParvOryx) is another example of a non-human host-specific virus currently under clinical investigation as an oncolytic vector. Safety and improved antitumor effects were shown in a glioblastoma trial [194,195], and H-1PV has now entered phase II against pancreatic cancer (NCT02653313). 

The amount and diversity of ongoing clinical trials for OVT is encouraging. Current developments in the filed especially highlight the importance of enhancing immunotherapeutic modes of action by means of vector engineering and combination treatment approaches. One such case follows in Section 8.

## 8. Mathematical Modeling of Oncolytic Virus Treatment with Immunotherapy

**Figure 5:**
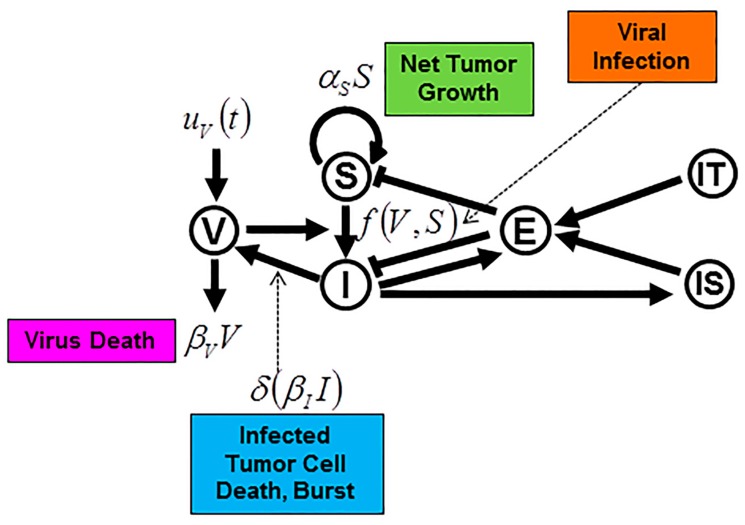
Explicit virus (V), and susceptible (S) and infected (I) cell model with addition of different immune system compartments (E, effector; IS, other immune system compartments such as antigen presenting cells) and immunotherapy (IT). Here, the effect of dendritic cell injection *IT* is modeled as an increase in effector cells. *U_V_*(*t*) describes the time-dependent introduction of the virus population. Second-order terms and interactions (death/exhaustion) left out for clarity. Note that the viral infection term $f\left(V,S\right)=\beta \frac{SV}{S+I}$ in [105]. See Figure 2b for comparison.

Immunotherapy can have different courses of action, including increasing the efficacy of antitumor immunity [196,197], reducing immune inhibitory mechanisms [198,199], or injection of dendritic cells [200] or engineered chimeric antigen receptor (CAR) T cells [201]. A variety of mathematical models have been developed to simulate different immunotherapies [202]. In 2015, Wares et al. modeled B16-F10 melanoma cells with adenovirus and dendritic cell injection in vivo [105]. A general scheme (Figure 5) is shown where dendritic cell injection as immune therapy (IT) is simulated as a direct increase in effector cells. 

Appropriate terms and parameters were used including susceptible cell net growth rate, death/exhaustion terms, infection, injections of therapy at specified time points, infected cells (4-1BBL from Ad-ΔB7/IL-12/4-1BBL) activating effector cells, infected cells (IL-12 from Ad-ΔB7/IL-12/4-1BBL) stimulating recruitment of antigen presenting cells (IS). Some terms or portions of terms are functions; for example, the T cell killing rate is expressed as a base T cell killing rate (*k*_0_) that can be enhanced (linearly, *c_k_*) by the presence of cytokine-producing infected cells (*I*), *k*(*I*) = *k*_0_ + *c_k_I*. The recruitments of T cells and APCs have a similar functional form of some constant, *c_N_*, multiplied by the number of infected cells (*I*) responsible for recruitment, *s_N_*(*I*) = *c_N_I*. Of note is that there is no explicit immune response modeled, just the interactions (recruitment) of certain immune components. Second, a conversion from tumor volume (mm^3^, experimental data) to number of cells is calculated by a conversion ratio of a volume of 1 mm^3^ to equal 1 million tumor cells. Lastly, all cell populations but uninfected (susceptible) tumor cells are initialized as being 0. Some model parameters are available in the literature including burst size [203], infected cell lysis rate [3,204,205,206], viral decay [207,208], T cell decay [209], maximum fractional T cell killing rate on tumor cells [210], and average time of an APCs to activate a T cell [211,212]. 

To calibrate unknown parameters with experimental data, a hierarchical approach was used. The control case was first simulated and fit to an exponential growth curve. The derived parameters were then kept constant while individually fitting for each of the individual therapies. Afterwards, those values were used to find parameters for the combination therapy. A Pearson’s *r* coefficient was calculated to give a rigorous fit, where values close to 1 indicates agreement. All models had a Pearson’s *r* greater than 0.98 except for the combination therapy model, which had a Pearson’s *r* = 0.92.

The Ad-ΔB7/IL-12/4-1BBL+DC model was used to predict alternative strategies: increasing dendritic cell dose, alternating Ad and DC injections, varying dose size, varying time between injections and number of doses. Within specifically posed biological and cytotoxic constraints, model simulations suggested that sequencing three injections of Ad followed by three injections of DC would be most effective to shrinking tumor volume. Further, the best order of Ad and DC injections and their respective optimal doses were highly dependent on temporal spacing. However, as appreciated by the authors, such predictions were likely to be model-dependent, emphasizing the need to simulate a variety of mathematical models with increasing and decreasing complexity to evaluate model predictive power and confidence in study conclusions. Model predictions that schedules that begin with viral injections would generally yield better decrease in tumor volume have been partially validated experimentally [213]. Interestingly, a recent robustness evaluation of the Wares model, using experimental data and a fitted mathematical model as inputs for Virtual Expansion of Populations for Analyzing Robustness of Therapies (VEPART) [214], also agrees with model predictions.

As the exact mechanisms and functional dependence of many of the different mechanisms in tumor–immune as well as virus–immune and virus–cancer interactions are yet to be fully deciphered, mathematical modeling in close dialog with experimental data may help identify likely and unlikely mechanistic properties of complex biological systems, which could and should iteratively inform subsequent experimental validation. 

## 9. Discussion and Future Directions

Despite the many breakthroughs in oncolytic virus biology and mathematical modeling, further improvement in several arenas is essential for successful implementation of OVT in clinical reality. Such research fields include the generation of safer and more potent vectors by genetic engineering, the development of high-capacity production pipelines and good manufacturing practice (GMP)-compliant facilities [215], the selection of appropriate oncolytic agents for certain applications by “build[ing] on the strengths of individual virus platforms” [216], and the design of more potent combination therapies [217].

The ideal route of delivery is among the currently most discussed questions in the field and can be exemplified by T-VEC and MV-NIS, which stand for intratumoral injection and systemic administration, respectively [217]. The approval of T-VEC was a huge achievement for the field that might either represent a stepping stone or a barrier for the approval of further oncolytics, which will have to be compared to this benchmark [217].

**Figure 6:**
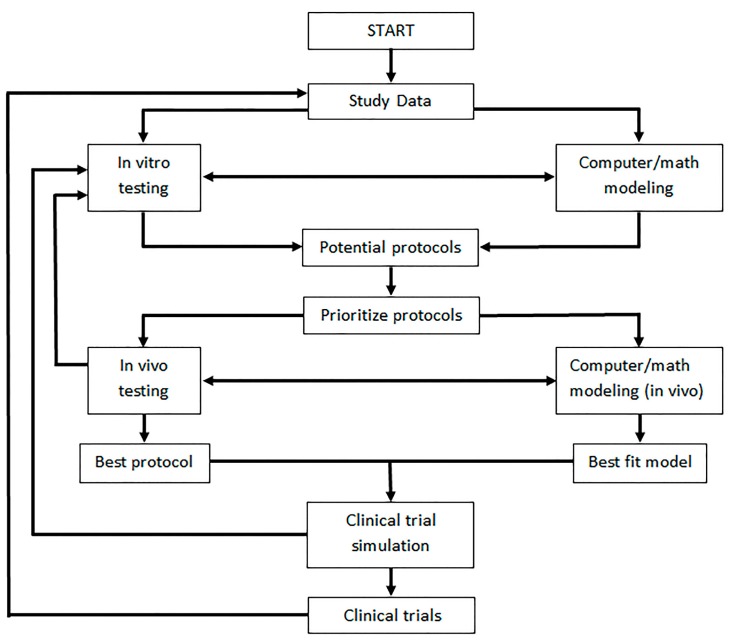
A workflow between experimental and computational data describing an iterative process among in vitro, in vivo, in silico, and clinical models, adapted from [218].

In a rapidly advancing field where various options for genetic modifications and treatment combinations are becoming available, the necessity for making rational choices of regimen combinations and scheduling becomes apparent. This can be supported by mathematical modeling as an in silico prediction tool for preclinical, experimental treatments and analyses of patient data in clinical trials. A “discovery workflow” has been discussed describing a working relationship between experimental and computational methods [218]. Between genetic engineering and the resulting effects on the immune system, a myriad of combination therapies involving oncolytic virus therapy is inevitable (and ongoing in clinical trials [219]). Oncolytic virus modeling (OVM) is a tool with proven predictive results when data-driven, which has been used to describe viral interactions with immune cells in different tissues and immune cells combined with immunotherapy. This may be a key fundamental feature for future modeling including personal immune profiles when accompanied with immune surveillance data. An iterative process wherein data continuously refines all models and vice versa could tackle the complexities of OVM (Figure 6). While ODE systems may be robust enough to describe spatial interactions, a PDE system may be used to elaborate on several mechanisms of action, developing a better understanding for an oncolytic virus and a specific cancer.

Collaborations between computational and experimental scientists will be instrumental for accurate and predictive in silico modeling for enhancing pre-clinical and eventually clinical efficacies. Examples of successful model development and data fitting have been highlighted herein; satisfactory model predictions, however, remain very much elusive. With sparse experimental data with low biological resolution of spatial and temporal scales of virus and cell dynamics as well as their interactions, mathematical formulations of different action terms may fit the data equally well but their prediction for system behavior in the future or under different experimental conditions may vary widely [104,220]. The integration of mathematical modeling into experimental cancer virology may help design experiments to collect longitudinal, high-resolution biological data to further our understanding of this complex, non-linear biological system. The expertise of several disciplines—immunology, virology, and cancer biology, to name a few—will guide appropriate experiments and allow for rational but shorter steps towards successful oncolytic virus therapies. Additionally, personalized medicine may be a natural extension of these models. The established concept of distinguishing important immune cells to generate an accurate mathematical model is a hopeful glimpse toward a patient-based description of the immune compartment in predicting cancer therapy.

## References

[1] Anderson A.R., Quaranta V. (2008). Integrative mathematical oncology. Nat. Rev. Cancer.

[2] Enderling H., Hlatky L., Hahnfeldt P. (2009). Migration rules: Tumours are conglomerates of self-metastases. Br. J. Cancer.

[3] Bajzer Z., Carr T., Josić K., Russell S.J., Dingli D. (2008). Modeling of cancer virotherapy with recombinant measles viruses. J. Theor. Biol..

[4] Eftimie R., Dushoff J., Bridle B., Bramson J.L., Earn D.J.D. (2011). Multi-stability and multi-instability phenomena in a mathematical model of tumor-immune-virus interactions. Bull. Math. Biol..

[5] Perelson A.S., Mirmirani M., Oster G.F. (1976). Optimal strategies in immunology. I. B-cell differentiation and proliferation. J. Math. Biol..

[6] Perelson A.S., Mirmirani M., Oster G.F. (1978). Optimal strategies in immunology. II. B memory cell production. J. Math. Biol..

[7] Perelson A.S., Goldstein B., Rocklin S. (1980). Optimal strategies in immunology III. The IgM-IgG switch. J. Math. Biol..

[8] Perelson A.S., Kirschner D.E., De Boer R. (1993). Dynamics of HIV infection of CD4^+^ T cells. Math. Biosci..

[9] Ho D.D., Neumann A.U., Perelson A.S., Chen W., Leonard J.M., Markowitz M. (1995). Rapid turnover of plasma virions and CD4 lymphocytes in HIV-1 infection. Nature.

[10] Perelson A.S., Neumann A.U., Markowitz M., Leonard J.M., Ho D.D. (1996). HIV-1 dynamics in vivo: Virion clearance rate, infected cell life-span, and viral generation time. Science.

[11] Perelson A.S., Essunger P., Cao Y., Vesanen M., Hurley A., Saksela K., Markowitz M., Ho D.D. (1997). Decay characteristics of HIV-1-infected compartments during combination therapy. Nature.

[12] Nowak M.A., May R. (2001). Viral Dynamics: Mathematical Principles of Immunology and Virology.

[13] Dock G. (1904). The influence of complicating diseases upon leukaemia. Am. J. Med. Sci..

[14] Hoster H.A., Zanes R.P., Von Haam E. (1949). Studies in Hodgkin’s syndrome; the association of viral hepatitis and Hodgkin’s disease; a preliminary report. Cancer Res..

[15] Southam C.M., Moore A.E. (1952). Clinical studies of viruses as antineoplastic agents with particular reference to Egypt 101 virus. Cancer.

[16] Georgiades J., Zielinski T., Cicholska A., Jordan E. (1959). Research on the oncolytic effect of APC viruses in cancer of the cervix uteri; preliminary report. Biul. Inst. Med. Mor. Gdansk.

[17] Asada T. (1974). Treatment of human cancer with mumps virus. Cancer.

[18] Cassady K.A., Haworth K.B., Jackson J., Markert J.M., Cripe T.P. (2016). To infection and beyond: The multi-pronged anti-cancer mechanisms of oncolytic viruses. Viruses.

[19] Araujo R.P., McElwain D.L. (2004). A history of the study of solid tumour growth: The contribution of mathematical modelling. Bull. Math. Biol..

[20] Byrne H., Preziosi L. (2003). Modelling solid tumour growth using the theory of mixtures. Math. Med. Biol..

[21] Folkman J., Hochberg M. (1973). Self-regulation of growth in three dimensions. J. Exp. Med..

[22] Vaupel P. (2004). The role of hypoxia-induced factors in tumor progression. Oncologist.

[23] Vaupel P., Harrison L. (2004). Tumor hypoxia: Causative factors, compensatory mechanisms, and cellular response. Oncologist.

[24] Deisboeck T.S., Wang Z. (2007). Cancer dissemination: A consequence of limited carrying capacity?. Med. Hypotheses.

[25] Hahnfeldt P., Panigrahy D., Folkman J., Hlatky L. (1999). Tumor development under angiogenic signaling: A dynamical theory of tumor growth, treatment response, and postvascular dormancy. Cancer Res..

[26] Gerlee P., Anderson A.R. (2015). The evolution of carrying capacity in constrained and expanding tumour cell populations. Phys. Biol..

[27] Benzekry S., Lamont C., Beheshti A., Tracz A., Ebos J.M., Hlatky L., Hahnfeldt P. (2014). Classical mathematical models for description and prediction of experimental tumor growth. PLoS Comput. Biol..

[28] Murphy H., Jaafari H., Dobrovolny H.M. (2016). Differences in predictions of ODE models of tumor growth: A cautionary example. BMC Cancer.

[29] Gerlee P. (2013). The model muddle: In search of tumor growth laws. Cancer Res..

[30] Brouwer A.F., Meza R., Eisenberg M.C. (2017). Parameter estimation for multistage clonal expansion models from cancer incidence data: A practical identifiability analysis. PLoS Comput. Biol..

[31] Eisenberg M.C., Robertson S.L., Tien J.H. (2013). Identifiability and estimation of multiple transmission pathways in cholera and waterborne disease. J. Theor. Biol..

[32] Eisenberg M.C., Hayashi M.A. (2014). Determining identifiable parameter combinations using subset profiling. Math. Biosci..

[33] Idema S., Dirven C.M.F., van Beusechem V.W., Carette J.E., Planque R., Noskel D.P., Lamfers M.L.M., Vandertop W.P. (2010). Objective determination of the oncolytic potency of conditionally-replicating adenoviruses using mathematical modeling. J. Gene Med..

[34] De Pillis L.G., Eladdadi A., Radunskaya A.E. (2014). Modeling cancer-immune responses to therapy. J. Pharmacokinet. Pharmacodyn..

[35] Wang Z., Butner J.D., Kerketta R., Cristini V., Deisboeck T.S. (2015). Simulating cancer growth with multiscale agent-based modeling. Semin. Cancer Biol..

[36] Wodarz D. (2016). Computational modeling approaches to the dynamics of oncolytic viruses. Wiley Interdiscip. Rev. Syst. Biol..

[37] Hanahan D., Weinberg R.A. (2011). Hallmarks of cancer: The next generation. Cell.

[38] Seymour L.W., Fisher K.D. (2016). Oncolytic viruses: Finally delivering. Br. J. Cancer.

[39] Martin G.S. (2001). The hunting of the Src. Nat. Rev. Mol. Cell Biol..

[40] Martin G.S. (2004). The road to Src. Oncogene.

[41] Sefton B.M., Hunter T., Raschke W.C. (1981). Evidence that the Abelson virus protein functions in vivo as a protein kinase that phosphorylates tyrosine. Proc. Natl. Acad. Sci. USA.

[42] Durst M., Gissmann L., Ikenberg H., zur Hausen H. (1983). A papillomavirus DNA from a cervical carcinoma and its prevalence in cancer biopsy samples from different geographic regions. Proc. Natl. Acad. Sci. USA.

[43] Munger K., Phelps W.C., Bubb V., Howley P.M., Schlegel R. (1989). The E6 and E7 genes of the human papillomavirus type 16 together are necessary and sufficient for transformation of primary human keratinocytes. J. Virol..

[44] Hacein-Bey-Abina S., Von Kalle C., Schmidt M., McCormack M.P., Wulffraat N., Leboulch P., Lim A., Osborne C.S., Pawliuk R., Morillon E. (2003). LMO2-associated clonal T cell proliferation in two patients after gene therapy for SCID-X1. Science.

[45] Takeda H., Takai A., Inuzuka T., Marusawa H. (2017). Genetic basis of hepatitis virus-associated hepatocellular carcinoma: Linkage between infection, inflammation, and tumorigenesis. J. Gastroenterol..

[46] Bluming A.Z., Ziegler J.L. (1971). Regression of Burkitt’s lymphoma in association with measles infection. Lancet.

[47] Cattaneo R., Miest T., Shashkova E.V., Barry M.A. (2008). Reprogrammed viruses as cancer therapeutics: Targeted, armed and shielded. Nat. Rev. Microbiol..

[48] Virgin S., Fields B.N., Knipe D.M., Howley P.M. (2007). Pathogenesis of Viral Infection. Fields’ Virology.

[49] Finkelshtein D., Werman A., Novick D., Barak S., Rubinstein M. (2013). LDL receptor and its family members serve as the cellular receptors for vesicular stomatitis virus. Proc. Natl. Acad. Sci. USA.

[50] Tatsuo H., Ono N., Tanaka K., Yanagi Y. (2000). SLAM (CDw150) is a cellular receptor for measles virus. Nature.

[51] Noyce R.S., Bondre D.G., Ha M.N., Lin L.T., Sisson G., Tsao M.S., Richardson C.D. (2011). Tumor cell marker PVRL4 (Nectin 4) is an epithelial cell receptor for measles virus. PLoS Pathog..

[52] Bankamp B., Takeda M., Zhang Y., Xu W., Rota P.A. (2011). Genetic characterization of measles vaccine strains. J. Infect. Dis..

[53] Dorig R.E., Marcil A., Chopra A., Richardson C.D. (1993). The human CD46 molecule is a receptor for measles virus (Edmonston strain). Cell.

[54] Naniche D., Varior-Krishnan G., Cervoni F., Wild T.F., Rossi B., Rabourdin-Combe C., Gerlier D. (1993). Human membrane cofactor protein (CD46) acts as a cellular receptor for measles virus. J. Virol..

[55] Nielsen L., Blixenkrone-Moller M., Thylstrup M., Hansen N.J., Bolt G. (2001). Adaptation of wild-type measles virus to CD46 receptor usage. Arch. Virol..

[56] Hammond A.L., Plemper R.K., Zhang J., Schneider U., Russell S.J., Cattaneo R. (2001). Single-chain antibody displayed on a recombinant measles virus confers entry through the tumor-associated carcinoembryonic antigen. J. Virol..

[57] Peng K.W., Holler P.D., Orr B.A., Kranz D.M., Russell S.J. (2004). Targeting virus entry and membrane fusion through specific peptide/MHC complexes using a high-affinity T-cell receptor. Gene Ther..

[58] Nakamura T., Peng K.W., Vongpunsawad S., Harvey M., Mizuguchi H., Hayakawa T., Cattaneo R., Russell S.J. (2004). Antibody-targeted cell fusion. Nat. Biotechnol..

[59] Cronin J., Zhang X.Y., Reiser J. (2005). Altering the tropism of lentiviral vectors through pseudotyping. Curr. Gene Ther..

[60] Funke S., Schneider I.C., Glaser S., Muhlebach M.D., Moritz T., Cattaneo R., Cichutek K., Buchholz C.J. (2009). Pseudotyping lentiviral vectors with the wild-type measles virus glycoproteins improves titer and selectivity. Gene Ther..

[61] Plattet P., Alves L., Herren M., Aguilar H.C. (2016). Measles virus fusion protein: Structure, function and inhibition. Viruses.

[62] Lamb R.A., Parks G.D., Fields B.N., Knipe D.M., Howley P.M. (2007). *Paramyxoviridae*: The Viruses and Their Replication. Fields’ Virology.

[63] Stegmann T., Hoekstra D., Scherphof G., Wilschut J. (1985). Kinetics of pH-dependent fusion between influenza virus and liposomes. Biochemistry.

[64] Van Rikxoort M., Michaelis M., Wolschek M., Muster T., Egorov A., Seipelt J., Doerr H.W., Cinatl J. (2012). Oncolytic effects of a novel influenza A virus expressing interleukin-15 from the NS reading frame. PLoS ONE.

[65] Kasloff S.B., Pizzuto M.S., Silic-Benussi M., Pavone S., Ciminale V., Capua I. (2014). Oncolytic activity of avian influenza virus in human pancreatic ductal adenocarcinoma cell lines. J. Virol..

[66] Pizzuto M.S., Silic-Benussi M., Ciminale V., Elderfield R.A., Capua I., Barclay W.S. (2016). An engineered avian-origin influenza A virus for pancreatic ductal adenocarcinoma virotherapy. J. Gen. Virol..

[67] Kalia M., Jameel S. (2011). Virus entry paradigms. Amino Acids.

[68] Marchini A., Bonifati S., Scott E.M., Angelova A.L., Rommelaere J. (2015). Oncolytic parvoviruses: From basic virology to clinical applications. Virol. J..

[69] Kirn D. (2015). Oncolytic Vaccinia Virus Cancer Therapy. U.S. Patent.

[70] Weller S.K., Coen D.M. (2012). Herpes simplex viruses: Mechanisms of DNA replication. Cold Spring Harb. Perspect. Biol..

[71] Moss B. (2013). Poxvirus DNA replication. Cold Spring Harb. Perspect. Biol..

[72] Hoeben R.C., Uil T.G. (2013). Adenovirus DNA replication. Cold Spring Harb. Perspect. Biol..

[73] Griffin D.E., Fields B.N., Knipe D.M., Howley P.M. (2007). Measles Virus. Fields’ Virology.

[74] Anacker D.C., Moody C.A. (2017). Modulation of the DNA damage response during the life cycle of human papillomaviruses. Virus Res..

[75] Longworth M.S., Laimins L.A. (2004). Pathogenesis of human papillomaviruses in differentiating epithelia. Microbiol. Mol. Biol. Rev..

[76] Petros P., Panagiotis T., Georgios I., Stefanos Z., Zacharoula K., Anastasia B., Georgios G., Simona V. (2016). Human papillomavirus’ life cycle and carcinogenesis. Maedica.

[77] Honess R.W., Roizman B. (1974). Regulation of herpesvirus macromolecular synthesis. I. Cascade regulation of the synthesis of three groups of viral proteins. J. Virol..

[78] Kakizoe Y., Nakaoka S., Beauchemin C.A., Morita S., Mori H., Igarashi T., Aihara K., Miura T., Iwami S. (2015). A method to determine the duration of the eclipse phase for in vitro infection with a highly pathogenic SHIV strain. Sci. Rep..

[79] Ruiz A.J., Russell S.J. (2015). MicroRNAs and oncolytic viruses. Curr. Opin. Virol..

[80] Bofill-De Ros X., Villanueva E., Fillat C. (2015). Late-phase miRNA-controlled oncolytic adenovirus for selective killing of cancer cells. Oncotarget.

[81] Leber M.F., Bossow S., Leonard V.H., Zaoui K., Grossardt C., Frenzke M., Miest T., Sawall S., Cattaneo R., von Kalle C. (2011). MicroRNA-sensitive oncolytic measles viruses for cancer-specific vector tropism. Mol. Ther..

[82] Springfeld C., von Messling V., Frenzke M., Ungerechts G., Buchholz C.J., Cattaneo R. (2006). Oncolytic efficacy and enhanced safety of measles virus activated by tumor-secreted matrix metalloproteinases. Cancer Res..

[83] Ketzer P., Kaufmann J.K., Engelhardt S., Bossow S., von Kalle C., Hartig J.S., Ungerechts G., Nettelbeck D.M. (2014). Artificial riboswitches for gene expression and replication control of DNA and RNA viruses. Proc. Natl. Acad. Sci. USA.

[84] José A., Rovira-Rigau M., Luna J., Giménez-Alejandre M., Vaquero E., de la García Torre B., Andreu D., Alemany R., Fillat C. (2014). A genetic fiber modification to achieve matrix-metalloprotease-activated infectivity of oncolytic adenovirus. J. Control. Release.

[85] Villanueva E., Navarro P., Rovira-Rigau M., Sibilio A., Méndez R., Fillat C. (2017). Translational reprogramming in tumour cells can generate oncoselectivity in viral therapies. Nat. Commun..

[86] Doerfler W. (2016). Beware of manipulations on the genome: Epigenetic destabilization through (foreign) DNA insertions. Epigenomics.

[87] Ranzani M., Annunziato S., Adams D.J., Montini E. (2013). Cancer gene discovery: Exploiting insertional mutagenesis. Mol. Cancer Res..

[88] Singh B.K., Li N., Mark A.C., Mateo M., Cattaneo R., Sinn P.L. (2016). Cell-to-cell contact and Nectin-4 govern spread of measles virus from primary human myeloid cells to primary human airway epithelial cells. J. Virol..

[89] Anderson B.D., Nakamura T., Russell S.J., Peng K.W. (2004). High CD46 receptor density determines preferential killing of tumor cells by oncolytic measles virus. Cancer Res..

[90] Rodriguez-Brenes I.A., Hofacre A., Fan H., Wodarz D. (2017). Complex dynamics of virus spread from low infection multiplicities: Implications for the spread of oncolytic viruses. PLoS Comput. Biol..

[91] Ali N., Haque M., Venturino E., Chakravarty S. (2017). Dynamics of a three species ratio-dependent food chain model with intra-specific competition within the top predator. Comput. Biol. Med..

[92] Siettos C.I., Russo L. (2013). Mathematical modeling of infectious disease dynamics. Virulence.

[93] Nowak M.A., Bangham C.R. (1996). Population dynamics of immune responses to persistent viruses. Science.

[94] Gatenby R.A. (1995). Models of tumor–host interaction as competing populations: Implications for tumor biology and treatment. J. Theor. Biol..

[95] Novozhilov A.S., Berezovskaya F.S., Koonin E.V., Karev G.P. (2006). Mathematical modeling of tumor therapy with oncolytic viruses: Regimes with complete tumor elimination within the framework of deterministic models. Biol. Direct.

[96] Perelson A.S., Ribeiro R.M. (2013). Modeling the within-host dynamics of HIV infection. BMC Biol..

[97] Arazi A., Pendergraft W.F., Ribeiro R.M., Perelson A.S., Hacohen N. (2013). Human systems immunology: Hypothesis-based modeling and unbiased data-driven approaches. Semin. Immunol..

[98] Canini L., Perelson A.S. (2014). Viral kinetic modeling: State of the art. J. Pharmacokinet. Pharmacodyn..

[99] Wodarz D. (2001). Viruses as antitumor weapons: Defining conditions for tumor remission. Cancer Res..

[100] Wodarz D. (2001). Mechanisms underlying antigen-specific CD8^+^ T cell homeostasis. Science.

[101] Dingli D., Offord C., Myers R., Peng K.W., Carr T.W., Josic K., Russell S.J., Bajzer Z. (2009). Dynamics of multiple myeloma tumor therapy with a recombinant measles virus. Cancer Gene Ther..

[102] Biesecker M., Kimn J.H., Lu H., Dingli D., Bajzer Z. (2010). Optimization of virotherapy for cancer. Bull. Math. Biol..

[103] Tian J.P. (2011). The replicability of oncolytic virus: Defining conditions in tumor virotherapy. Math. Biosci. Eng..

[104] Wodarz D., Komarova N. (2009). Towards predictive computational models of oncolytic virus therapy: Basis for experimental validation and model selection. PLoS ONE.

[105] Wares J.R., Crivelli J.J., Yun C.O., Choi I.K., Gevertz J.L., Kim P.S. (2015). Treatment strategies for combining immunostimulatory oncolytic virus therapeutics with dendritic cell injections. Math. Biosci. Eng..

[106] Lichty B.D., Breitbach C.J., Stojdl D.F., Bell J.C. (2014). Going viral with cancer immunotherapy. Nat. Rev. Cancer.

[107] Workenhe S.T., Mossman K.L. (2014). Oncolytic virotherapy and immunogenic cancer cell death: Sharpening the sword for improved cancer treatment strategies. Mol. Ther..

[108] Barik S. (2016). What Really Rigs Up RIG-I?. J. Innate Immun..

[109] Beug S.T., Tang V.A., LaCasse E.C., Cheung H.H., Beauregard C.E., Brun J., Nuyens J.P., Earl N., St-Jean M., Holbrook J. (2014). Smac mimetics and innate immune stimuli synergize to promote tumor death. Nat. Biotechnol..

[110] Bhat R., Rommelaere J. (2015). Emerging role of natural killer cells in oncolytic virotherapy. Immunotargets Ther..

[111] Mellman I., Coukos G., Dranoff G. (2011). Cancer immunotherapy comes of age. Nature.

[112] Kim Y., Clements D.R., Sterea A.M., Jang H.W., Gujar S.A., Lee P.W. (2015). Dendritic cells in oncolytic virus-based anti-cancer therapy. Viruses.

[113] Mosmann T.R., Cherwinski H., Bond M.W., Giedlin M.A., Coffman R.L. (1986). Two types of murine helper T cell clone. I. Definition according to profiles of lymphokine activities and secreted proteins. J. Immunol..

[114] Farrar J.D., Asnagli H., Murphy K.M. (2002). T helper subset development: Roles of instruction, selection, and transcription. J. Clin. Investig..

[115] Flossdorf M., Rossler J., Buchholz V.R., Busch D.H., Hofer T. (2015). CD8^+^ T cell diversification by asymmetric cell division. Nat. Immunol..

[116] Hand T.W., Kaech S.M. (2009). Intrinsic and extrinsic control of effector T cell survival and memory T cell development. Immunol. Res..

[117] Wang N., Liang H., Zen K. (2014). Molecular mechanisms that influence the macrophage M1–M2 polarization balance. Front. Immunol..

[118] Darrasse-Jeze G., Deroubaix S., Mouquet H., Victora G.D., Eisenreich T., Yao K.H., Masilamani R.F., Dustin M.L., Rudensky A., Liu K. (2009). Feedback control of regulatory T cell homeostasis by dendritic cells in vivo. J. Exp. Med..

[119] Tuve S., Liu Y., Tragoolpua K., Jacobs J.D., Yumul R.C., Li Z.Y., Strauss R., Hellström K.E., Disis M.L., Roffler S. (2009). In situ adenovirus vaccination engages T effector cells against cancer. Vaccine.

[120] Workenhe S.T., Pol J.G., Lichty B.D., Cummings D.T., Mossman K.L. (2013). Combining oncolytic HSV-1 with immunogenic cell death-inducing drug mitoxantrone breaks cancer immune tolerance and improves therapeutic efficacy. Cancer Immunol. Res..

[121] Grossardt C., Engeland C.E., Bossow S., Halama N., Zaoui K., Leber M.F., Springfeld C., Jaeger D., von Kalle C., Ungerechts G. (2013). Granulocyte-macrophage colony-stimulating factor-armed oncolytic measles virus is an effective therapeutic cancer vaccine. Hum. Gene Ther..

[122] Veinalde R., Grossardt C., Hartmann L., Bourgeois-Daigneault M.C., Bell J.C., Jäger D., von Kalle C., Ungerechts G., Engeland C.E. (2017). Oncolytic measles virus encoding interleukin-12 mediates potent antitumor effects through T cell activation. Oncoimmunology.

[123] Engeland C.E., Grossardt C., Veinalde R., Bossow S., Lutz D., Kaufmann J.K., Shevchenko I., Umansky V., Nettelbeck D.M., Weichert W. (2014). CTLA-4 and PD-L1 checkpoint blockade enhances oncolytic measles virus therapy. Mol. Ther..

[124] Zamarin D., Holmgaard R.B., Ricca J., Plitt T., Palese P., Sharma P., Merghoub T., Wolchok J.D., Allison J.P. (2017). Intratumoral modulation of the inducible co-stimulator ICOS by recombinant oncolytic virus promotes systemic anti-tumour immunity. Nat. Commun..

[125] Yu F., Wang X., Guo Z.S., Bartlett D.L., Gottschalk S.M., Song X.T. (2014). T-cell engager-armed oncolytic vaccinia virus significantly enhances antitumor therapy. Mol. Ther..

[126] Fajardo C.A., Guedan S., Rojas L.A., Moreno R., Arias-Badia M., de Sostoa J., June C.H., Alemany R. (2017). Oncolytic adenoviral delivery of an EGFR-targeting T-cell engager improves antitumor efficacy. Cancer Res..

[127] Freedman J.D., Hagel J., Scott E.M., Psallidas I., Gupta A., Spiers L., Miller P., Kanellakis N., Ashfield R., Fisher K.D. (2017). Oncolytic adenovirus expressing bispecific antibody targets T-cell cytotoxicity in cancer biopsies. EMBO Mol. Med..

[128] Vigil A., Martinez O., Chua M.A., García-Sastre A. (2008). Recombinant Newcastle disease virus as a vaccine vector for cancer therapy. Mol. Ther..

[129] Bridle B.W., Stephenson K.B., Boudreau J.E., Koshy S., Kazdhan N., Pullenayegum E., Brunellière J., Bramson J.L., Lichty B.D., Wan Y. (2010). Potentiating cancer immunotherapy using an oncolytic virus. Mol. Ther..

[130] Pol J.G., Zhang L., Bridle B.W., Stephenson K.B., Rességuier J., Hanson S., Chen L., Kazdhan N., Bramson J.L., Stojdl D.F. (2014). Maraba virus as a potent oncolytic vaccine vector. Mol. Ther..

[131] Kaufman H.L., Taback B., Sherman W., Kim D.W., Shingler W.H., Moroziewicz D., DeRaffele G., Mitcham J., Carroll M.W., Harrop R. (2009). Phase II trial of modified vaccinia Ankara (MVA) virus expressing 5T4 and high dose interleukin-2 (IL-2) in patients with metastatic renal cell carcinoma. J. Transl. Med..

[132] Puzanov I., Milhem M.M., Minor D., Hamid O., Li A., Chen L., Chastain M., Gorski K.S., Anderson A., Chou J. (2016). Talimogene laherparepvec in combination with ipilimumab in previously untreated, unresectable stage IIIB-IV melanoma. J. Clin. Oncol..

[133] Zamarin D., Holmgaard R.B., Subudhi S.K., Park J.S., Mansour M., Palese P., Merghoub T., Wolchok J.D., Allison J.P. (2014). Localized oncolytic virotherapy overcomes systemic tumor resistance to immune checkpoint blockade immunotherapy. Sci. Transl. Med..

[134] Huang B., Sikorski R., Kirn D.H., Thorne S.H. (2011). Synergistic anti-tumor effects between oncolytic vaccinia virus and paclitaxel are mediated by the IFN response and HMGB1. Gene Ther..

[135] Russell S.J., Peng K.W., Bell J.C. (2012). Oncolytic virotherapy. Nat. Biotechnol..

[136] Jha B.K., Dong B., Nguyen C.T., Polyakova I., Silverman R.H. (2013). Suppression of antiviral innate immunity by sunitinib enhances oncolytic virotherapy. Mol. Ther..

[137] Bossow S., Grossardt C., Temme A., Leber M.F., Sawall S., Rieber E.P., Cattaneo R., von Kalle C., Ungerechts G. (2011). Armed and targeted measles virus for chemovirotherapy of pancreatic cancer. Cancer Gene Ther..

[138] Abate-Daga D., Andreu N., Camacho-Sánchez J., Alemany R., Herance R., Millán O., Fillat C. (2011). Oncolytic adenoviruses armed with thymidine kinase can be traced by PET imaging and show potent antitumoural effects by ganciclovir dosing. PLoS ONE.

[139] Breitbach C.J., Arulanandam R., De Silva N., Thorne S.H., Patt R., Daneshmand M., Moon A., Ilkow C., Burke J., Hwang T.H. (2013). Oncolytic vaccinia virus disrupts tumor-associated vasculature in humans. Cancer Res..

[140] Liu T.C., Hwang T., Park B.H., Bell J., Kirn D.H. (2008). The targeted oncolytic poxvirus JX-594 demonstrates antitumoral, antivascular, and anti-HBV activities in patients with hepatocellular carcinoma. Mol. Ther..

[141] Arulanandam R., Batenchuk C., Angarita F.A., Ottolino-Perry K., Cousineau S., Mottashed A., Burgess E., Falls T.J., De Silva N., Tsang J. (2015). VEGF-mediated induction of PRD1-BF1/Blimp1 expression sensitizes tumor vasculature to oncolytic virus infection. Cancer Cell.

[142] Bose A., Taylor J.L., Alber S., Watkins S.C., Garcia J.A., Rini B.I., Ko J.S., Cohen P.A., Finke J.H., Storkus W.J. (2011). Sunitinib facilitates the activation and recruitment of therapeutic anti-tumor immunity in concert with specific vaccination. Int. J. Cancer.

[143] Monod J. (1949). The Growth of Bacterial Cultures. Ann. Rev. Microbiol..

[144] Michaelis L., Menten M.L., Johnson K.A., Goody R.S. (2011). The original Michaelis constant: Translation of the 1913 Michaelis-Menten paper. Biochemistry.

[145] Wodarz D., Chan C.N., Trinité B., Komarova N.L., Levy D.N. (2014). On the laws of virus spread through cell populations. J. Virol..

[146] Komarova N.L., Anghelina D., Voznesensky I., Trinité B., Levy D.N., Wodarz D. (2013). Relative contribution of free-virus and synaptic transmission to the spread of HIV-1 through target cell populations. Biol. Lett..

[147] Jacobsen K., Pilyugin S.S. (2015). Analysis of a mathematical model for tumor therapy with a fusogenic oncolytic virus. Math. Biosci..

[148] Aref S., Bailey K., Fielding A. (2016). Measles to the rescue: A review of oncolytic measles virus. Viruses.

[149] Atienza J.M., Zhu J., Wang X., Xu X., Abassi Y. (2005). Dynamic monitoring of cell adhesion and spreading on microelectronic sensor arrays. J. Biomol. Screen..

[150] Crivelli J.J., Foldes J., Kim P.S., Wares J.R. (2012). A mathematical model for cell cycle-specific cancer virotherapy. J. Biol. Dyn..

[151] Fonseca G.J., Thillainadesan G., Yousef A.F., Ablack J.N., Mossman K.L., Torchia J., Mymryk J.S. (2012). Adenovirus evasion of interferon-mediated innate immunity by direct antagonism of a cellular histone posttranslational modification. Cell Host Microbe.

[152] Herschke F., Plumet S., Duhen T., Azocar O., Druelle J., Laine D., Wild T.F., Rabourdin-Combe C., Gerlier D., Valentin H. (2007). Cell-cell fusion induced by measles virus amplifies the type I interferon response. J. Virol..

[153] Roediger E.K., Kugathasan K., Zhang X., Lichty B.D., Xing Z. (2008). Heterologous boosting of recombinant adenoviral prime immunization with a novel vesicular stomatitis virus-vectored tuberculosis vaccine. Mol. Ther..

[154] Bridle B.W., Boudreau J.E., Lichty B.D., Brunellière J., Stephenson K., Koshy S., Bramson J.L., Wan Y. (2009). Vesicular stomatitis virus as a novel cancer vaccine vector to prime antitumor immunity amenable to rapid boosting with adenovirus. Mol. Ther..

[155] Reis C.L., Pacheco J.M., Ennis M.K., Dingli D. (2010). In silico evolutionary dynamics of tumour virotherapy. Integr. Biol..

[156] Wu J.T., Byrne H.M., Kirn D.H., Wein L.M. (2001). Modeling and analysis of a virus that replicates selectively in tumor cells. Bull. Math. Biol..

[157] Friedman A., Tao Y. (2003). Analysis of a model of a virus that replicates selectively in tumor cells. J. Math. Biol..

[158] Bateman A.R., Harrington K.J., Kottke T., Ahmed A., Melcher A.A., Gough M.J., Linardakis E., Riddle D., Dietz A., Lohse C.M. (2002). Viral fusogenic membrane glycoproteins kill solid tumor cells by nonapoptotic mechanisms that promote cross presentation of tumor antigens by dendritic cells. Cancer Res..

[159] Kavanová L., Matiašková K., Levá L., Štěpánová H., Nedbalcová K., Matiašovic J., Faldyna M., Salát J. (2017). Concurrent infection with porcine reproductive and respiratory syndrome virus and *Haemophilus parasuis* in two types of porcine macrophages: Apoptosis, production of ROS and formation of multinucleated giant cells. Vet. Res..

[160] Kozak R.A., Hattin L., Biondi M.J., Corredor J.C., Walsh S., Xue-Zhong M., Manuel J., McGilvray I.D., Morgenstern J., Lusty E. (2017). Replication and oncolytic activity of an avian orthoreovirus in human hepatocellular carcinoma cells. Viruses.

[161] Soomro M.H., Shi R., She R., Yang Y., Wang T., Wu Q., Li H., Hao W. (2017). Molecular and structural changes related to hepatitis E virus antigen and its expression in testis inducing apoptosis in Mongolian gerbil model. J. Viral Hepat..

[162] Du J., Wang L., Wang Y., Shen J., Pan C., Meng Y., Yang C., Ji H., Dong W. (2016). Autophagy and apoptosis induced by Chinese giant salamander (*Andrias davidianus*) iridovirus (CGSIV). Vet. Microbiol..

[163] Reshi L., Wu H.C., Wu J.L., Wang H.V., Hong J.R. (2016). GSIV serine/threonine kinase can induce apoptotic cell death via p53 and pro-apoptotic gene Bax upregulation in fish cells. Apoptosis.

[164] Chen X.Y., Wen C.M., Wu J.L., Su Y.C., Hong J.R. (2016). Giant seaperch iridovirus (GSIV) induces mitochondria-mediated cell death that is suppressed by bongkrekic acid and cycloheximide in a fish cell line. Virus Res..

[165] Nardacci R., Perfettini J.L., Grieco L., Thieffry D., Kroemer G., Piacentini M. (2015). Syncytial apoptosis signaling network induced by the HIV-1 envelope glycoprotein complex: An overview. Cell Death Dis..

[166] ClinicalTrials.gov. https://clinicaltrials.gov/.

[167] Kelly E., Russell S.J. (2007). History of oncolytic viruses: Genesis to genetic engineering. Mol. Ther..

[168] Liu B.L., Robinson M., Han Z.Q., Branston R.H., English C., Reay P., McGrath Y., Thomas S.K., Thornton M., Bullock P. (2003). ICP34.5 deleted herpes simplex virus with enhanced oncolytic, immune stimulating, and anti-tumour properties. Gene Ther..

[169] Hughes T., Coffin R.S., Lilley C.E., Ponce R., Kaufman H.L. (2014). Critical analysis of an oncolytic herpesvirus encoding granulocyte-macrophage colony stimulating factor for the treatment of malignant melanoma. Oncolytic Virother..

[170] Andtbacka R.H., Kaufman H.L., Collichio F., Amatruda T., Senzer N., Chesney J., Delman K.A., Spitler L.E., Puzanov I., Agarwala S.S. (2015). Talimogene laherparepvec improves durable response rate in patients with advanced melanoma. J. Clin. Oncol..

[171] Kohlhapp F.J., Kaufman H.L. (2016). Molecular Pathways: Mechanism of Action for Talimogene Laherparepvec, a New Oncolytic Virus Immunotherapy. Clin. Cancer Res..

[172] Kirn B., Starc V. (2007). Continuous axial contraction wave in the free wall of the guinea pig left ventricle. Comput. Biol. Med..

[173] Kirn D.H., Thorne S.H. (2009). Targeted and armed oncolytic poxviruses: A novel multi-mechanistic therapeutic class for cancer. Nat. Rev. Cancer.

[174] Parato K.A., Breitbach C.J., Le Boeuf F., Wang J., Storbeck C., Ilkow C., Diallo J.S., Falls T., Burns J., Garcia V. (2012). The oncolytic poxvirus JX-594 selectively replicates in and destroys cancer cells driven by genetic pathways commonly activated in cancers. Mol. Ther..

[175] Heo J., Reid T., Ruo L., Breitbach C.J., Rose S., Bloomston M., Cho M., Lim H.Y., Chung H.C., Kim C.W. (2013). Randomized dose-finding clinical trial of oncolytic immunotherapeutic vaccinia JX-594 in liver cancer. Nat. Med..

[176] Dispenzieri A., Tong C., LaPlant B., Lacy M.Q., Laumann K., Dingli D., Zhou Y., Federspiel M.J., Gertz M.A., Hayman S. (2017). Phase I trial of systemic administration of Edmonston strain of measles virus genetically engineered to express the sodium iodide symporter in patients with recurrent or refractory multiple myeloma. Leukemia.

[177] Galanis E., Atherton P.J., Maurer M.J., Knutson K.L., Dowdy S.C., Cliby W.A., Haluska P., Long H.J., Oberg A., Aderca I. (2015). Oncolytic measles virus expressing the sodium iodide symporter to treat drug-resistant ovarian cancer. Cancer Res..

[178] Dingli D., Peng K.W., Harvey M.E., Greipp P.R., O’Connor M.K., Cattaneo R., Morris J.C., Russell S.J. (2004). Image-guided radiovirotherapy for multiple myeloma using a recombinant measles virus expressing the thyroidal sodium iodide symporter. Blood.

[179] Myers R.M., Greiner S.M., Harvey M.E., Griesmann G., Kuffel M.J., Buhrow S.A., Reid J.M., Federspiel M., Ames M.M., Dingli D. (2007). Preclinical pharmacology and toxicology of intravenous MV-NIS, an oncolytic measles virus administered with or without cyclophosphamide. Clin. Pharmacol. Ther..

[180] Lech P.J., Pappoe R., Nakamura T., Tobin G.J., Nara P.L., Russell S.J. (2014). Antibody neutralization of retargeted measles viruses. Virology.

[181] Russell S.J., Federspiel M.J., Peng K.W., Tong C., Dingli D., Morice W.G., Lowe V., O’Connor M.K., Kyle R.A., Leung N. (2014). Remission of disseminated cancer after systemic oncolytic virotherapy. Mayo Clin. Proc..

[182] Russell S.J. Measles as a versatile oncolytic agent. Proceedings of the International Meeting on Replicating Oncolytic Virus Therapeutics.

[183] Croyle M.A., Callahan S.M., Auricchio A., Schumer G., Linse K.D., Wilson J.M., Brunner L.J., Kobinger G.P. (2004). PEGylation of a vesicular stomatitis virus G pseudotyped lentivirus vector prevents inactivation in serum. J. Virol..

[184] Hudacek A.W., Navaratnarajah C.K., Cattaneo R. (2013). Development of measles virus-based shielded oncolytic vectors: Suitability of other paramyxovirus glycoproteins. Cancer Gene Ther..

[185] Miest T.S., Yaiw K.C., Frenzke M., Lampe J., Hudacek A.W., Springfeld C., von Messling V., Ungerechts G., Cattaneo R. (2011). Envelope-chimeric entry-targeted measles virus escapes neutralization and achieves oncolysis. Mol. Ther..

[186] Evgin L., Ilkow C.S., Bourgeois-Daigneault M.C., de Souza C.T., Stubbert L., Huh M.S., Jennings V.A., Marguerie M., Acuna S.A., Keller B.A. (2016). Complement inhibition enables tumor delivery of LCMV glycoprotein pseudotyped viruses in the presence of antiviral antibodies. Mol. Ther. Oncolytics.

[187] Lilly C.L., Villa N.Y., de Lemos Matos A., Ali H.M., Dhillon J.S., Hofland T., Rahman M.M., Chan W., Bogen B., Cogle C., McFadden G. (2017). Ex vivo oncolytic virotherapy with myxoma virus arms multiple allogeneic bone marrow transplant leukocytes to enhance graft versus tumor. Mol. Ther. Oncolytics.

[188] Ong H.T., Federspiel M.J., Guo C.M., Ooi L.L., Russell S.J., Peng K.W., Hui K.M. (2013). Systemically delivered measles virus-infected mesenchymal stem cells can evade host immunity to inhibit liver cancer growth. J. Hepatol..

[189] Ong H.T., Hasegawa K., Dietz A.B., Russell S.J., Peng K.W. (2007). Evaluation of T cells as carriers for systemic measles virotherapy in the presence of antiviral antibodies. Gene Ther..

[190] Mader E.K., Maeyama Y., Lin Y., Butler G.W., Russell H.M., Galanis E., Russell S.J., Dietz A.B., Peng K.W. (2009). Mesenchymal stem cell carriers protect oncolytic measles viruses from antibody neutralization in an orthotopic ovarian cancer therapy model. Clin. Cancer Res..

[191] Brun J., McManus D., Lefebvre C., Hu K., Falls T., Atkins H., Bell J.C., McCart J.A., Mahoney D., Stojdl D.F. (2010). Identification of genetically modified Maraba virus as an oncolytic rhabdovirus. Mol. Ther..

[192] Travassos da Rosa A.P., Tesh R.B., Travassos da Rosa J.F., Herve J.P., Main A.J. (1984). Carajas and Maraba viruses, two new vesiculoviruses isolated from phlebotomine sand flies in Brazil. Am. J. Trop. Med. Hyg..

[193] Zamarin D., Pesonen S. (2015). Replication-competent viruses as cancer immunotherapeutics: Emerging clinical data. Hum. Gene Ther..

[194] Geletneky K., Huesing J., Rommelaere J., Schlehofer J.R., Leuchs B., Dahm M., Krebs O., von Knebel Doeberitz M., Huber B., Hajda J. (2012). Phase I/IIa study of intratumoral/intracerebral or intravenous/intracerebral administration of parvovirus H-1 (ParvOryx) in patients with progressive primary or recurrent glioblastoma multiforme: ParvOryx01 protocol. BMC Cancer.

[195] Geletneky K., Weiss C., Bernhard H., Capper D., Leuchs B., Marchini A., Rommelaere J. (2016). Atim-29. First clinical observation of improved anti-tumor effects of viro-immunotherapy with oncolytic parvovirus H-1 in combination with PD-1 checkpoint blockade and bevacicumab in patients with recurrent glioblastoma. Neuro Oncol..

[196] Zhou J., Xi Y., Mu X., Zhao R., Chen H., Zhang L., Wu Y., Li Q. (2017). Antitumor immunity induced by VE-cadherin modified DC vaccine. Oncotarget.

[197] Escribà-Garcia L., Alvarez-Fernández C., Tellez-Gabriel M., Sierra J., Briones J. (2017). Dendritic cells combined with tumor cells and α-galactosylceramide induce a potent, therapeutic and NK-cell dependent antitumor immunity in B cell lymphoma. J. Transl. Med..

[198] Nolan E., Savas P., Policheni A.N., Darcy P.K., Vaillant F., Mintoff C.P., Dushyanthen S., Mansour M., Pang J.B., Fox S.B. (2017). Combined immune checkpoint blockade as a therapeutic strategy for BRCA1-mutated breast cancer. Sci. Transl. Med..

[199] Dammeijer F., Lievense L.A., Kaijen-Lambers M.E., van Nimwegen M., Bezemer K., Hegmans J.P., van Hall T., Hendriks R.W., Aerts J.G. (2017). Depletion of tumor-associated macrophages with a CSF-1r kinase inhibitor enhances antitumor immunity and survival induced by DC immunotherapy. Cancer Immunol. Res..

[200] Huang J.H., Zhang S.N., Choi K.J., Choi I.K., Kim J.H., Lee M.G., Lee M., Kim H., Yun C.O. (2010). Therapeutic and tumor-specific immunity induced by combination of dendritic cells and oncolytic adenovirus expressing IL-12 and 4-1BBL. Mol. Ther..

[201] Nishio N., Diaconu I., Liu H., Cerullo V., Caruana I., Hoyos V., Bouchier-Hayes L., Savoldo B., Dotti G. (2014). Armed oncolytic virus enhances immune functions of chimeric antigen receptor-modified T cells in solid tumors. Cancer Res..

[202] Walker R., Enderling H. (2016). From concept to clinic: Mathematically informed immunotherapy. Curr. Probl. Cancer.

[203] Chen Y., DeWeese T., Dilley J., Zhang Y., Li Y., Ramesh N., Lee J., Pennathur-Das R., Radzyminski J., Wypych J. (2001). CV706, a prostate cancer-specific adenovirus variant, in combination with radiotherapy produces synergistic antitumor efficacy without increasing toxicity. Cancer Res..

[204] Friedman A., Tian J.P., Fulci G., Chiocca E.A., Wang J. (2006). Glioma virotherapy: Effects of innate immune suppression and increased viral replication capacity. Cancer Res..

[205] Ganly I., Mautner V., Balmain A. (2000). Productive replication of human adenoviruses in mouse epidermal cells. J. Virol..

[206] Jogler C., Hoffmann D., Theegarten D., Grunwald T., Uberla K., Wildner O. (2006). Replication properties of human adenovirus in vivo and in cultures of primary cells from different animal species. J. Virol..

[207] Li H.L., Li S., Shao J.Y., Lin X.B., Cao Y., Jiang W.Q., Liu R.Y., Zhao P., Zhu X.F., Zeng M.S. (2008). Pharmacokinetic and pharmacodynamic study of intratumoral injection of an adenovirus encoding endostatin in patients with advanced tumors. Gene Ther..

[208] Wang Y., Wang H., Li C.Y., Yuan F. (2006). Effects of rate, volume, and dose of intratumoral infusion on virus dissemination in local gene delivery. Mol. Cancer Ther..

[209] De Boer R.J., Oprea M., Antia R., Murali-Krishna K., Ahmed R., Perelson A.S. (2001). Recruitment times, proliferation, and apoptosis rates during the CD8^+^ T-cell response to lymphocytic choriomeningitis virus. J. Virol..

[210] Del Vecchio M., Bajetta E., Canova S., Lotze M.T., Wesa A., Parmiani G., Anichini A. (2007). Interleukin-12: Biological properties and clinical application. Clin. Cancer Res..

[211] Van Stipdonk M.J., Lemmens E.E., Schoenberger S.P. (2001). Naïve CTLs require a single brief period of antigenic stimulation for clonal expansion and differentiation. Nat. Immunol..

[212] Veiga-Fernandes H., Walter U., Bourgeois C., McLean A., Rocha B. (2000). Response of naïve and memory CD8^+^ T cells to antigen stimulation in vivo. Nat. Immunol..

[213] Zhang S.N., Choi I.K., Huang J.H., Yoo J.Y., Choi K.J., Yun C.O. (2011). Optimizing DC vaccination by combination with oncolytic adenovirus coexpressing IL-12 and GM-CSF. Mol. Ther..

[214] Barish S., Ochs M.F., Sontag E.D., Gevertz J.L. (2017). Evaluating optimal therapy robustness by virtual expansion of a sample population, with a case study in cancer immunotherapy. Proc. Natl. Acad. Sci. USA.

[215] Ungerechts G., Bossow S., Leuchs B., Holm P.S., Rommelaere J., Coffey M., Coffin R., Bell J., Nettelbeck D.M. (2016). Moving oncolytic viruses into the clinic: Clinical-grade production, purification, and characterization of diverse oncolytic viruses. Mol. Ther. Methods Clin. Dev..

[216] Breitbach C.J., Lichty B.D., Bell J.C. (2016). Oncolytic viruses: Therapeutics with an identity crisis. EBioMedicine.

[217] Russell S.J., Peng K.W. (2017). Oncolytic virotherapy: A contest between apples and oranges. Mol. Ther..

[218] McGuire M.F., Enderling H., Wallace D.I., Batra J., Jordan M., Kumar S., Panetta J.C., Pasquier E. (2013). Formalizing an integrative, multidisciplinary cancer therapy discovery workflow. Cancer Res..

[219] Friedman G.K., Markert J.M., Gillespie G.Y. (2017). Combination strategies enhance oncolytic virotherapy. Oncotarget.

[220] Komarova N.L., Wodarz D. (2013). Virus dynamics in the presence of synaptic transmission. Math. Biosci..

